# 2809. In Vitro Synergy of Antibiotic Combinations Against *Enterococcus gallinarum*

**DOI:** 10.1093/ofid/ofad500.2420

**Published:** 2023-11-27

**Authors:** David Landman, Meir Cherniak, Jacob Strahilevitz

**Affiliations:** Hadassah Hebrew University Medical Center, Jerusalem, Yerushalayim, Israel; Hadassah Hebrew University Medical Center, Jerusalem, Yerushalayim, Israel; Hadassah Hebrew University Medical Center, Jerusalem, Yerushalayim, Israel

## Abstract

**Background:**

Enterococci are an important cause of endocarditis, particularly involving prosthetic valves. The prevalence of *E. gallinarum* has been increasing in recent years. Unlike *E. faecalis*, no information is available regarding the bactericidal activity of ampicillin alone or combined with gentamicin or ceftriaxone against *E. gallinarum*.

**Methods:**

Ten blood culture isolates of *E. gallinarum* were studied. The isolates came from unique patients at a single hospital. MICs of ampicillin, ceftriaxone and gentamicin were done by the CLSI microdilution method. ATCC strains *E. faecalis* 29212 and *P. aeruginosa* 27853 were used as controls. Time-kill studies were performed in CA-MHB for ampicillin 10 mg/L, ceftriaxone 10 mg/L, gentamicin at 0.25 x MIC up to a maximum of 4 mg/L, ampicillin + ceftriaxone, and ampicillin + gentamicin. Bactericidal activity was defined as a 1,000-fold decrease in the log_10_ cfu/mL at 24 h. Synergy was defined as a 100-fold decrease in the log_10_ cfu/ml at 24 h in a combination compared to each single agent.

**Results:**

The MICs of ampicillin ranged from 1 to 8 mg/L, gentamicin from 2 to 16 mg/L, and ceftriaxone were all ≥ 32 mg/L. Results of the time-kill studies are summarized in the Table. Ampicillin alone was bactericidal against a single isolate. For that isolate, testing of ampicillin at 0.25 x MIC with gentamicin demonstrated synergy. The combination of ampicillin + ceftriaxone was synergistic against a single isolate, but was otherwise similar to ampicillin alone. Ampicillin + gentamicin was bactericidal against all isolates and was synergistic against all but one isolate.

**Conclusion:**

Ampicillin alone was generally not bactericidal against *E. gallinarum* but had synergistic bactericidal activity when combined with gentamicin, similar to the findings with *E. faecalis*. However, unlike *E. faecalis*, ampicillin + ceftriaxone was not bactericidal against most isolates of *E. gallinarum*. These findings support the suggestion that ampicillin + gentamicin might be the optimal treatment for endovascular infections caused by *E. gallinarum*.

**Disclosures:**

**All Authors**: No reported disclosures

**Table:** 

Antibiotic	Change log_10_ cfu/mL at 4 h	Change log_10_ cfu/mL at 24 h	Number bactericidal	Number with synergy
Ampicillin	-0.5 ± 0.8	-2.0 ± 1.1	1/10	
Ampicillin + Ceftriaxone	-0.1 ± 1.2	-2.2 ± 1.7	3/10	1/10
Ampicillin + Gentamicin	-3.6 ± 1.3	-4.9 ± 0.3	10/10	9/10

